# Rhizosphere element circling, multifunctionality, aboveground productivity and trade-offs are better predicted by rhizosphere rare taxa

**DOI:** 10.3389/fpls.2022.985574

**Published:** 2022-09-08

**Authors:** Puchang Wang, Leilei Ding, Chao Zou, Yujun Zhang, Mengya Wang

**Affiliations:** ^1^School of Life Sciences, Guizhou Normal University, Guiyang, China; ^2^Guizhou Institute of Prataculture, Guizhou Academy of Agricultural Sciences, Guiyang, China; ^3^College of Animal Science, Guizhou University, Guiyang, China

**Keywords:** abundant taxa, rare taxa, diversity, trade-off, multifunctionality

## Abstract

Microbes, especially abundant microbes in bulk soils, form multiple ecosystem functions, which is relatively well studied. However, the role of rhizosphere microbes, especially rhizosphere rare taxa vs. rhizosphere abundant taxa in regulating the element circling, multifunctionality, aboveground net primary productivity (ANPP) and the trade-offs of multiple functions remains largely unknown. Here, we compared the multiple ecosystem functions, the structure and function of rhizosphere soil bacterial and fungal subcommunities (locally rare, locally abundant, regionally rare, regionally abundant, and entire), and the role of subcommunities in the *Zea mays* and *Sophora davidii* sole and *Z. mays/S. davidii* intercropping ecosystems in subtropical China. Results showed that intercropping altered multiple ecosystem functions individually and simultaneously. Intercropped *Z. mays* significantly decreased the trade-off intensity compared to sole *Z. mays*, the trade-off intensity under intercropped *S. davidii* was significantly higher than under intercropped *Z. mays*. The beta diversities of bacterial and fungal communities, and fungal functions in each subcommunity significantly differed among groups. Network analysis showed intercropping increased the complexity and positive links of rare bacteria in *Z. mays* rhizosphere, but decreased the complexity and positive links of rare bacteria in *S. davidii* rhizosphere and the complexity and positive links of fungi in both intercropped plants rhizosphere. Mantel test showed significant changes in species of locally rare bacteria were most strongly related to nitrogen-cycling multifunctionality, ANPP and trade-offs intensity, significant changes in species of locally rare fungus were most strongly related to carbon-cycling multifunctionality, phosphorus-cycling multifunctionality, and average ecosystem multifunctionality. This research highlights the potential and role of rare rhizosphere microorganisms in predicting and regulating system functions, productivity, and trade-offs.

## Introduction

Losses in taxonomic and functional diversities are pervasively at both global and local scales (Radchuk et al., 2016; Baldrighi et al., 2017; Huang et al., 2019). This trend is predicted to continue over this century (Huang et al., 2019), and raises increasing concerns on the influence of biodiversity on ecosystem functions (Karolína et al., 2014; Radchuk et al., 2016; Baldrighi et al., 2017). Unlike flora (Niklaus et al., 2017; Wang et al., 2020b) or fauna (Gagic et al., 2015; Tonin et al., 2018) both which have been well studied in the studies of the relationship between biodiversity and ecosystem function, soil microorganisms represent the richest and highest diverse life (Delgado-Baquerizo et al., 2017; Luo et al., 2018; Chen et al., 2020a), nevertheless, the relationships between soil microorganisms and ecosystem functions are not fully understood (Delgado-Baquerizo et al., 2017). Previous studies have found that soil microbes contribute to driving multiple ecosystem functions simultaneously (MF) (Fanin et al., 2017; Jiao et al., 2018; Chen et al., 2020a), such as carbon and nutrient cycling (Delgado-Baquerizo et al., 2017; Jiao et al., 2018) and productivity (George et al., 2019; Zheng et al., 2019). However, most studies have centered on the temperate communities (Guerra et al., 2020) and the bulk soil communities (Luo et al., 2018; Wen et al., 2020). In contrast, subtropical data on soil microbial diversity and ecosystem functions are particularly scant (Guerra et al., 2020). Furthermore, the rhizosphere is a true hot point of plant-microbial-soil interactions, and the rhizosphere microbial communities are clearly distinctive from the surrounding bulk soil’s (Fan et al., 2017; Jiao et al., 2018), because plants filter microbes for special structures and function (Del Galdo Jos et al., 2003), to benefit their growth, nutrition (Mendes et al., 2014), and function (Lu et al., 2018). Notwithstanding, how rhizosphere bacterial and fungal communities participate in the nutrient cycling of rhizosphere soil, and drive the rhizosphere multiple functions, aboveground primary productivity and trade-offs among functions remains less explicitly acknowledged. Although bacteria and fungi are the most frequently studied communities in soil biodiversity and ecosystem function research (Guerra et al., 2020), their diversity effects on ecosystem functions have not yet been fully explored for the rhizosphere soils.

Besides, the majority of studies were based on abundant taxa of microbiome (Chen et al., 2020a; Liang et al., 2020). A large number of low abundance taxa (rare microbial taxa) were deleted before analyzing data of microbiome (Lynch and Neufeld, 2015; Jousset et al., 2017; Chen et al., 2020a). However, there is a theoretical contradiction for this deletion. On the one hand, the influential mass ratio hypothesis states (Grime, 1998) that the influences of species/functions on an ecosystem function/process is in proportion to their biomass/relative abundance (Karolína et al., 2014; Bagousse-Pingueta et al., 2019), in this sense, it may be reasonable to focus only on the role of abundant taxa. On the other hand, the most of taxa in almost all ecosystems are low abundance (rare) (David et al., 2013; Jain et al., 2014; Lynch and Neufeld, 2015), and dominant species account for most of the total abundance (Dee et al., 2019), resulting in high diversity in the rare subcommunity (Jain et al., 2014; Lynch and Neufeld, 2015) and low diversity in the dominant subcommunity. Generally, the selection effect [selection/occurrence of some particular species (or functions)/identity effects] and complementary effect (niche partitioning/different resource utilization/facilitation) are recognized mechanisms to interpret the role of biodiversity in shaping ecosystem functions (Loreau, 2000; Mensah et al., 2020; Ding and Wang, 2021). Since high species diversity should increase the likelihood of the selection effect (Mensah et al., 2020) and/or the complementary effect (Mensah et al., 2018), rare subcommunities or their functions is inferred to contribute in greater proportion to a given ecosystem function than the dominant. This contradicts the mass ratio hypothesis. Besides, the deletion changed substantially the profile of rare taxa (Chen et al., 2020a). Unfortunately, rare species are often more sensitive (Jain et al., 2014; Guo et al., 2020; Zhou et al., 2020b) and vulnerable to vanish firstly (David et al., 2013). Therefore, if the above inference is true, conservation and use of rare species will be more imperative than those of the abundance species to maintain ecosystem functions. However, the ecological role of rare species is poorly known (Säterberg et al., 2019). Although the complementarity and selection effects contribute to MF, their relative contributions remains controversial (Mensah et al., 2020). Moreover, the relative importance of these two effects of rare rhizosphere microbes in explaining rhizosphere functions, plant productivity and trade-offs are understudied.

In this study, our hypothesis was that rare rhizosphere taxa contributed to multiple functions and trade-offs in larger proportion than the abundant taxa did. To examine this hypothesis, we characterized the rhizosphere abundant and rare bacteria and fungi, rhizosphere C/N/P-cycling multifunctionality, ecosystem multifunctionality, aboveground net primary productivity and trade-offs, as well as explored how bacteria and fungi with different abundance differently link to the multiple rhizosphere functions, aboveground net primary productivity and trade-offs in subtropical sole and intercropping systems in China.

## Materials and methods

### Study area and design

Sampling was conducted in a cropping common garden in the Guizhou Academy of Agricultural Sciences (26°30′N, 106°39′E, 1,100 m a.s.l.). This region undergoes a north subtropical monsoon climate characterized by annual precipitation of 1,130 mm, annual average air temperature of 15.3°C, and Haplic alisol soil. The sole and intercropping systems were selected based on the fact that monoculture systems can reduce biodiversity (Shao et al., 2020), intercropping systems can enhance biodiversity (Martin-Guay et al., 2018; Dingha et al., 2021), and both systems are widely used around the world. The experimental design was a randomized block design (Luo et al., 2018) established using three cropping systems, including *Sophora davidii* (Franch.) monocropping (MS), *Zea mays* L. monocropping (MZ), and intercropping of both (IS and IZ), in May 1st 2019. Five repeated plots (2 m × 5 m per repeat) were arranged for each system with intervals of 1 m. MS was planted with row distances and plant distances of 0.6 m, MZ was planted with row distances of 0.6 m and plant distance of 0.3 m. Row and plant distances of *S. davidii* (Franch.) and *Z. mays* L. in the intercropping system were identical to in monocropping system and the distance between adjacent rows was 0.3 m in the intercropping system. Since this small-scale design can minimize the variation in either of none-design factors, such as topographies, climate, and soil type (Chen et al., 2020c; Ding and Wang, 2021), this approach is convenient to compare the contributions of diversity vs. other drivers in shaping ecosystem function (van der Plas, 2019), allowing the resolution required to capture relationships between the plant-selected microbes and the rhizosphere element circling, aboveground productivity and trade-offs.

Fresh aboveground *S. davidii* and *Z. mays* were harvested using a 1 m^2^ (1 × 1) quadrat in the monoculture and intercropping plots, respectively, in August 11th 2019. After the fresh was killed at 105°C and oven-dried at 65°C, the ANPP was obtained. Soil that tightly adhering to roots (rhizosphere soils) was collected by trembling the roots (Xiong et al., 2020), and was mixed to obtain a composite sample for each replicate. The soil was sieved using 2-mm meshes. A total of 20 composite samples [(two monocropping + two intercropping) × five replicates] were obtained. The sample was divided into subsamples for downstream analysis.

### Individual rhizosphere functions

The rhizosphere physicochemical properties and C, N, and P-circling enzyme activities were assayed using the methods listed in previous studies (Zheng et al., 2019; Ding et al., 2020a,b) and the Supplementary Description of Method. Briefly, rhizosphere soil pH was measured with a suspension (soil: water = 1:2.5 w/v); Water content (WC, %) was obtained by oven-drying at 105°C (Wang et al., 2022). Rhizosphere C-circling functions were characterized as follows (Bowker et al., 2013; Bastida et al., 2016; Luo et al., 2018; Ding et al., 2020a): organic carbon (g kg^−1^, OC) was assayed using potassium dichromate volumetric method, β-glucosidase (C-circling enzyme, μmol d^−1^ g^−1^ dry soil, βG) was detected using an ELISA test kit (Shanghai Enzyme-linked Biotechnology Co., Ltd., China). Microbial biomass carbon (mg kg^−1^, MBC) was determined by the fumigation–extraction method. Rhizosphere N-circling functions were characterized as follows (Jiao et al., 2018; Luo et al., 2018; Bagousse-Pingueta et al., 2019; Ding et al., 2020a): ammoniacal nitrogen (mg kg^−1^, NH4_N) was assayed using ultraviolet spectrophotometry, nitrate nitrogen (mg kg^−1^, NO3_N) was using the colorimetric method based on extraction of potassium chloride, inorganic nitrogen (mg kg^−1^, IN) was the sum of ammoniacal and nitrate nitrogen, five N-circling enzymes [*N*-acetylglucosaminidase (NAG); Leucine aminopeptidase (LAP), Nitrogenase, Nitric oxide synthetase (NOS), Glutamine synthetase (GS); μmol d^−1^ g^−1^ dry soil] were detected using ELISA kits. Microbial biomass nitrogen (mg kg^−1^, MBN) was determined by the fumigation–extraction method. Rhizosphere P-circling functions were characterized as follows (Bowker et al., 2013; Bastida et al., 2016; Jiao et al., 2018; Luo et al., 2018; Bagousse-Pingueta et al., 2019; Ding et al., 2020a): total phosphorus (mg kg^−1^, TP) was determined by NaOH digestion, available phosphorus (mg kg^−1^, AP) was assayed via the NaHCO_3_-ultraviolet spectrometer, and acid phosphatase (P-circling enzyme, μmol d^−1^ g^−1^ dry soil, ACP) was detected using ELISA test kits. These properties were used because they either measure real functions or are good surrogates of C, N, and P-cycling (Bowker et al., 2013; Delgado-Baquerizo et al., 2017; Bagousse-Pingueta et al., 2019; Chen et al., 2020a,b).

### DNA extraction and PCR amplification

Genomic DNA was extracted using HiPure Soil DNA Kits (Guangzhou Meiji Biotechnology Co., Ltd., China) following the manufacturer’s instructions. The quality of extracted DNA was checked with electrophoresis in a 1.2% (wt/vol) agarose gel and a NanoDrop 2000 spectrophotometer (Thermo Fisher Scientific, United States). The V3–V4 region of the bacterial 16S rRNA gene and the internal transcribed spacer (ITS) regions of the fungal rRNA gene were amplified with primers 341F and 806R and ITS3_KYO2 and ITS4, respectively (detailed PCR conditions are described in Supplementary Description of Method).

### Amplicon sequencing and data processing

Amplicons were extracted from 2% agarose gels (Gong et al., 2021) and purified using the AxyPrep DNA Gel Extraction Kit (Axygen Biosciences, Union City, CA, United States) according to the manufacturer’s instructions and quantified using ABI StepOnePlus Real-Time PCR System (Life Technologies, Foster, CA, United States). Purified amplicons were pooled in equimolar and paired-end sequenced (PE250) on an Illumina platform by Guangzhou Genedenovo Biotechnology Co., Ltd. (Guangzhou, China). The raw data were deposited in NCBI (Accession number: PRJNA731978 for 16S, PRJNA731989 for ITS).

Raw data were processed as follows: briefly, reads containing more than 10% of unknown nucleotides and containing less than 50% of bases with quality (*Q*-value) > 20 were removed using FASTP (V 0.18.0^1^). Paired-end clean reads were merged as raw tags using FLSAH (V 1.2.11^2^) with a minimum overlap of 10 bp and mismatch error rates of 2%. The unique tag sequence was selected using Mothur (v1.39.1^3^). Chimeras were removed using UCHIME algorithm in USEARCH^4^. Sequences were then assigned to operational taxonomic unit (OTUs) at a 97% similarity threshold (Ding et al., 2020a; Ding and Wang, 2021; Jin et al., 2022b) using UPARSE (USEARCH v9.2.64, see Text Footnote 4). The representative OTU sequences were classified into organisms by a naive Bayesian model using RDP classifier (version 2.2) based on SILVA database (version 132^5^) for bacteria or UNITE database (version 8.0^6^) for fungi. Bioinformatics analysis was done using an online platform^7^ by Guangzhou Genedenovo Biotechnology Co., Ltd. (Guangzhou, China).

### Definition of abundant and rare microbial taxa

The locally abundant and rare OTUs were defined using abundance thresholds of 0.1 and 0.01% in a sample (Lynch and Neufeld, 2015), and regionally abundant and rare OTUs were defined by abundance thresholds of 0.01 and 0.001% across samples (Mo et al., 2018). The subsequent analyses were done at five levels: whole, locally abundant, locally rare, regionally abundant, and regionally rare OTUs. The richness, Simpson diversity, and Shannon diversity indices were applied to describe the taxonomic diversity.

### Predicted functions of bacteria and fungi

Tax4Fun and FAPROTAX 1.2.3 were used to predict bacterial functional profiles (Aßhauer et al., 2015; Louca et al., 2016). FUNGuild (Guilds_v1.1) was used to ppredicting fungal functional profiles (Nguyen et al., 2016). The ‘‘Vegan’’ package was applied to calculate the richness, Shannon diversity, and Simpson diversity of bacterial and fungal functions at the commonly used level (i.e., Tax4Fun KEGG 2 level and FUNGuild Guild level) in R v3.5.3^8^.

### Multifunctionality and trade-off intensity

Multifunctionality is a crucial management and ecological index (Delgado-Baquerizo et al., 2020), and is defined as the synthesis of different ecosystem properties at a small scale (Bagousse-Pingueta et al., 2019; Ding and Wang, 2021). Rhizosphere C/N/P-circling multifunctionality (CCMF, NCMF, and PCMF) was calculated based on rhizosphere C/N/P-circling functions (Garibotti et al., 2018) in the Section “Individual rhizosphere functions,” and rhizosphere multifunctionality was calculated based on all rhizosphere functions (Bagousse-Pingueta et al., 2019) listed in Section “Individual rhizosphere functions.” *Z*-score transformation was performed to standardize the data of microbial community and ecosystem functions (Bastida et al., 2016; Bagousse-Pingueta et al., 2019) using the “standardizeZScore” function (Byrnes et al., 2014) in R before calculation of multifunctionality (Chen et al., 2020a). Average approach (the mean of all standardized functions) is intuitive and easily interpretable and widely used in the multifunctionality studies (Delgado-Baquerizo et al., 2016, 2017), therefore, average approaches-based multifunctionality index for each sample were calculated (Shi et al., 2021). Trade-off intensity was defined as the geometric distance from a point to a straight line of 1:1 to quantify the trade-off intensity between two properties of ecosystem (Zhong et al., 2020).

### Data analysis

The statistical analysis followed the work flow and methods in previous studies (Ding et al., 2020a; Ding and Wang, 2021) and in the Supplementary Description of Method. The Shapiro–Wilk normality test and Levene’s test were used to test the normality and homoscedasticity of the data, respectively (Gao et al., 2021). When the data could meet the normality and homoscedasticity criteria simultaneously, ANOVA and *t*-test were used to test the significance of difference among groups. When the data could not meet, Kruskal–Wallis rank sum test and Wilcoxon test were used. The ηp^2^ (partial eta-squared) statistic was conducted to test the relative influences of plant identity (*S. davidii* and *Z. mays*), system type (sole and intercropping) and interaction on each individual rhizosphere functions in IBM SPSS (version 25, IBM, Armonk, NY, United States). Principal component analysis (PCA) (Merino-Martín et al., 2021) was used to determine which functions are the main ecosystem functions in the rhizosphere. The ANOSIM (Analysis of Similarities) test with 9,999 permutations was used to determine significant differences in rhizosphere ecosystem functions between plant species (*S. davidii* and *Z. mays*), system type (sole and intercropping) and among groups (MZ, IZ, MS, and IS) and in species/functions among groups for each subcommunity. Correlation relationships among individual rhizosphere ecosystem functions were visualized based Spearman using “igraph” package (Csardi and Nepusz, 2006). To retain the number of edges, vertices, and positive edges of microbial association networks, robust correlations were built based absolute value of Pearson correlation’s *r* > 0.8 and false discovery rate-corrected *p* < 0.001 using ‘‘WGCNA’’ and ‘‘igraph’’ packages in R. Gephi 0.9.2^9^ were applied to visualize the networks.

Non-metric multidimensional scaling (NMDS) (Jiao et al., 2017; Khashi u Rahman et al., 2021) was applied to simplify samples or OUTs in high-dimensional to low-dimensional space for location using the “metaMDS” function based on the Bray–Curtis distance (He et al., 2021). Linear discriminant analysis (LDA) effect size (LEfSe) was run to determine the significantly enriched clades (LDA scores ≥ 2 and *p* < 0.05).

According to the definition of the selection effect (Mensah et al., 2020), we used Spearman rank correlations (Zheng et al., 2019) to select the species, functions, and diversity of the whole community, and abundant and rare subcommunities that were significantly related to C/N/P-circling multifunctionality (CCMF, NCMF, and PCMF), average ecosystem multifunctionality (AEMF), and aboveground net primary productivity (ANPP), respectively. We used Kruskal–Wallis rank sum test to select the species, functions, and diversity of the whole, abundant, and rare subcommunities that significantly changed among groups. We then calculated the intersection of species/functions/diversities that significantly changed and were significantly related to the above ecosystem functions. To decipher how microbes link multifunctionality (Delgado-Baquerizo et al., 2017), the Mantel test (Ding et al., 2020a) with 9,999 permutations was performed to detect the correlation between the intersection and above ecosystem functions, based on the Spearman method and Euclidean distance matrix. The greater the correlation of the Mantel test, the greater the potential impact intensity of species/functions/diversities on the above ecosystem functions (Jiao et al., 2017; Mo et al., 2018; Zheng et al., 2019; Zhou et al., 2019b; Xiong et al., 2020; Xue et al., 2020; Ding and Wang, 2021). The “ggplot2” (Wickham, 2016) and “circlize” (Gu, 2014) packages were used to visualize the results.

## Results

### Intercropping altered individual functions, multifunctionality, aboveground net primary productivity, and trade-offs

**FIGURE 1:**
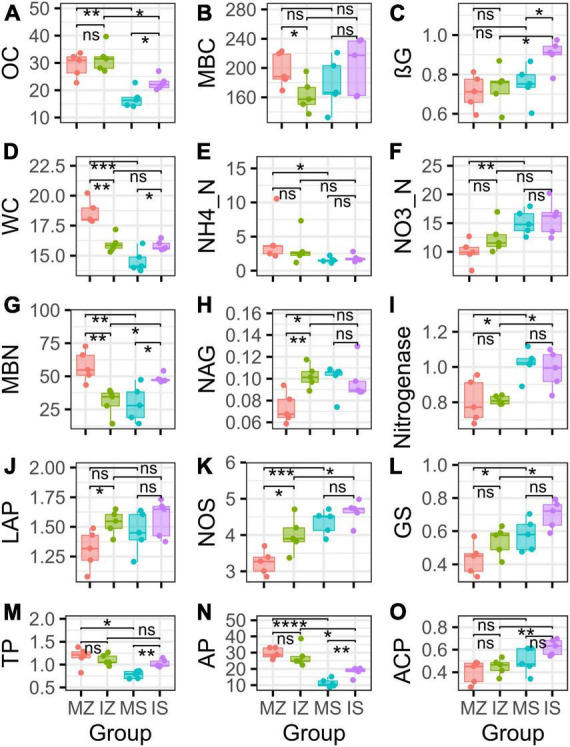
Intercropping altered multiple rhizosphere functions. “ns,” *p* > 0.05; “*,” *p* < 0.05; “**,” *p* < 0.01; “***,” *p* < 0.001; MZ, monoculture *Zea mays*; IZ, intercropping *Zea mays*; MS, monoculture *Sophora davidii*; IZ, intercropping *Sophora davidii*. **(A)** Intercropping altered the organic carbon (OC), **(B)** intercropping altered the microbial biomass carbon content (MBC), **(C)** intercropping altered the β-glucosidase activity (βG), **(D)** intercropping altered the water content (WC), **(E)** intercropping altered the ammoniacal nitrogen content (NH4_N), **(F)** intercropping altered the nitrate nitrogen content (NO3_N), **(G)** intercropping altered the microbial biomass nitrogen content (MBN), **(H)** intercropping altered the *N*-cetylglucosaminidase activity (NAG), **(I)** intercropping altered Nitrogenase activity, **(J)** intercropping altered the Leucine aminopeptidase activity (LAP), **(K)** intercropping altered the Nitric oxide synthetase activity (NOS), **(L)** intercropping altered the Glutamine synthetase activity (GS), **(M)** intercropping altered the total phosphorus content (TP), **(N)** intercropping altered the available phosphorus content (AP), and **(O)** intercropping altered the acid phosphatase activity (ACP).

Compared with *S. davidii*, regardless of system type, the remarkably higher rhizosphere OC of *Z. mays* was observed (71% higher under monoculture, *t*-test *p* < 0.05; 40% higher under intercropping, *t*-test *p* < 0.05, Figure 1A). Compared with monoculture, intercropped *Z. mays* did not significantly change OC, intercropped *S. davidii* significantly elevated OC by 33% (*t*-test *p* < 0.05). Regardless of system type, statistical difference was not observed in MBC between *Z. mays* and *S. davidii* (*t*-test *p* > 0.05), (Figure 1B). Compared with under sole *S. davidii*, MBC under intercropping has increasing trend (by 14%, *t*-test *p* > 0.05), but MBC under intercropped *Z*. *mays* significantly decreased by 17% (*t*-test *p* < 0.05). Compared with monoculture, intercropped *Z. mays* did not significantly change the βG activity in rhizosphere (*t*-test *p* > 0.05, Figure 1C), but intercropped *S. davidii* increased the βG activity by 20% (*t*-test *p* < 0.05), therefore, despite statistical difference was not found in the βG activity between sole *Z. mays* and *S. davidii* (*t*-test *p* > 0.05), the βG activity under intercropped *S. davidii* was higher 23% than under intercropped *Z. mays* (*t*-test *p* < 0.05). WC was higher under sole *S. davidii* than under sole *Z. mays* by 22% (*t*-test *p* < 0.05, Figure 1D). Compared with monoculture, intercropped *Z. mays* decreased WC by 14% (*t*-test *p* < 0.05), intercropped *S. davidii* increased WC by 9% (*t*-test *p* < 0.05), resulting in insignificant difference in WC between intercropped *S. davidii* and *Z. mays* (*t*-test *p* > 0.05). NH4_N was higher under sole *Z. mays* than under sole *S. davidii* by 189% (Wilcoxon test *p* < 0.05, Figure 1E), NO3_N was higher under sole *S. davidii* than under sole *Z. mays* by 53% (*t*-test *p* < 0.05, Figure 1F), statistical difference was not detected between two plants, and between sole and intercropping (*t*-test *p* > 0.05). MBN was lower under sole *S. davidii* than under sole *Z. mays* by 49% (*t*-test *p* < 0.05, Figure 1G), but NAG, Nitrogenase, NOS, and GS under sole *S. davidii* were higher than under sole *Z. mays* by 36, 26, 34, and 33%, respectively (*t*-test *p* < 0.05, Figures 1H,I,K,L), the LAP activity under sole *S. davidii* were higher than under sole *Z. mays* by 12% (*t*-test *p* > 0.05, Figure 1J). Compared with monoculture, intercropped *Z. mays* decreased MBN by 47% (*t*-test *p* > 0.05), but increased NAG, LAP, and NOS by 40, 18, and 23%, respectively (*t*-test *p* < 0.05). Intercropped *S. davidii* increased MBN by 65% (*t*-test *p* > 0.05). MBN, Nitrogenase, NOS, and GS under intercropped *S. davidii* were higher than under intercropped *Z. mays* by 59, 21, 16, and 30%, respectively (*t*-test *p* < 0.05). TP and AP under sole *Z. mays* were higher than under sole *S. davidii* by 50 and 163%, respectively (*t*-test *p* < 0.05, Figures 1M,N). Intercropped *Z. mays* did not significantly change TP, AP, and ACP, in comparison to sole *Z. mays* (*t*-test *p* < 0.05, Figures 1M–O), intercropped *S. davidii* increased TP and AP in comparison to sole *S. davidii* (*t*-test *p* < 0.05), and statistical difference was not found in TP between intercropped *S. davidii* and *Z. mays* (*t*-test *p* > 0.05). AP under intercropped Sophora davidii was lower than under intercropped *Z. mays* (*t*-test *p* < 0.05), ACP under intercropped *S. davidii* was higher than under intercropped *Z. mays* (*t*-test *p* < 0.05). Statistical differences were not observed in IN and pH among group (Supplementary Figure 1, *t*-test *p* > 0.05).

**FIGURE 2:**
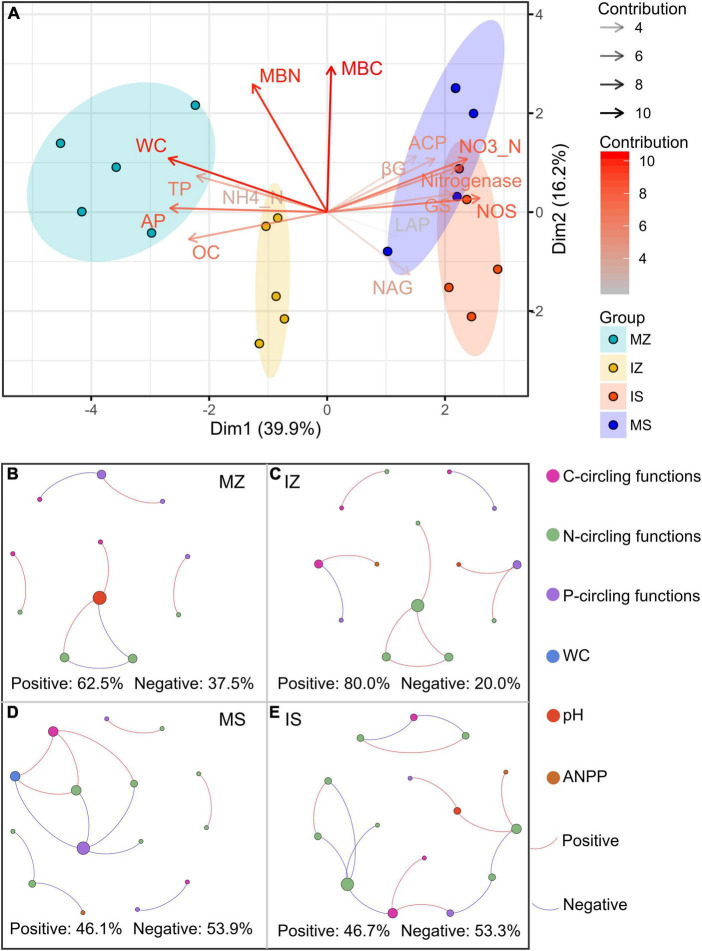
**(A)** Principal component analysis of multiple rhizosphere functions and in the different groups, showing overall intuitive distribution of multiple functions and aboveground net primary productivity, and the contribution of each function to this distribution. **(B–E)** Relationships among individual rhizosphere functions and aboveground net primary productivity (ANPP). MZ, monoculture *Zea mays*; IZ, intercropping *Zea mays*; MS, monoculture *Sophora davidii*; IZ, intercropping *Sophora davidii*.

**FIGURE 3:**
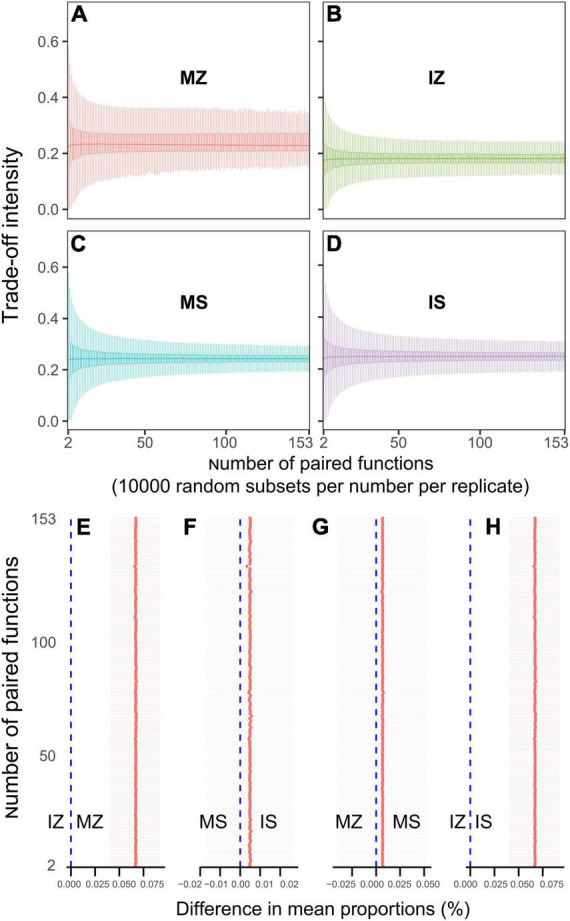
**(A–D)** Boxplots indicating that the trade-off intensity of functions changed with the number of paired functions. **(E–H)** Difference of the trade-off intensity between groups at the number of paired functions (Wilcoxon test at α = 0.05). MZ, monoculture *Zea mays*; IZ, intercropping *Zea mays*; MS, monoculture *Sophora davidii*; IZ, intercropping *Sophora davidii*.

**FIGURE 4:**
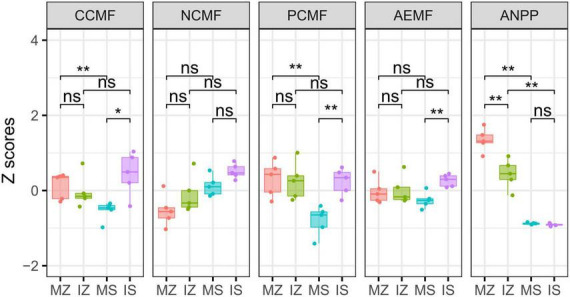
Intercropping altered rhizosphere C, N, P circling multifunctionality (CCMF, NCMF, and PCMF), average rhizosphere ecosystem multifunctionality (AEMF) and aboveground net primary productivity (ANPP). “ns,” *p* > 0.05; “*,” *p* < 0.05; “**,” *p* < 0.01; MZ, monoculture *Zea mays*; IZ, intercropping *Zea mays*; MS, monoculture *Sophora davidii*; IZ, intercropping *Sophora davidii*.

The ηp^2^ statistic showed that plant species was the main factor that impacted the OC, βG, NH4_N, NO3_N, Nitrogenase, NOS, GS, TP, AP, ACP, WC, and ANPP, and system type was the main factor that impacted the LAP, the interaction between plant species and system type was the main factor that impacted the MBC, MBN, and NAG (Supplementary Table 1). PCA showed that sampling points were well separated between plant species (ANOSIM *r* = 0.41, *p* = 0.046) and among groups (ANOSIM *r* = 0.38, *p* < 0.001), but sampling points were not separated between system type (ANOSIM *r* = 0.01, *p* = 0.352), indicating that plant species was the main factor that impacted the rhizosphere functions (Figure 2A). The main contributors to these separations were WC, MBN, AP, and MBC. The sole and intercropped *Z. mays* had higher WC, TP, AP, and OC, the sole and intercropped *S. davidii* had higher NO3_N, Nitrogenase, NOS, and GS. Correlation network analysis showed that intercropping increased the complexity of functional relationships and the frequency of functional synergy, while decreased the frequency of functional trade-off compared to monoculture (Figures 2B–E). The trade-off analysis showed that the intensity of ecosystem function trade-off was highly variable when low number of paired functions was included, but tends to be stable as the number of included paired functions increased, indicated that it is necessary to consider sufficient number of function pairs when evaluating the strength of function trade-offs (Figures 3A–D). Wilcoxon test showed that intercropped *Z. mays* significantly decreased the trade-off intensity compared to sole *Z. mays*, trade-off intensity under intercropped *S. davidii* was significantly higher than under intercropped *Z. mays* (Figures 3E–H). Multifunctionality analysis showed intercropping significantly increased the CCMF, and PCMF and AEMF (*p* = 0.032, 0.0079, and 0.0079) of the *S. davidii* rhizosphere; however, it did not change the NCMF and ANPP of *S. davidii* (Wilcoxon test, *p* = 0.056 – 0.22, Figure 4). Additionally, intercropping significantly decreased the ANPP of *Z. mays* (Wilcoxon test, *p* = 0.008), but did not alter the CCMF, NCMF, and PCMF and AEMF of *Z. mays* rhizosphere (Wilcoxon test, *p* = 0.15 – 0.84, Figure 4).

### Intercropping shifted the diversity of rhizosphere microbes

The average Good coverage for all samples was >98.7%, suggesting that the sampling was adequate (Jin et al., 2022a). Intercropping made whole bacterial richness, whole bacterial Shannon diversity, locally abundant bacterial Simpson diversity, regionally rare bacterial richness, regionally rare bacterial Shannon diversity, regionally abundant fungal richness evidently distinct between two plants (ANOVA *p* < 0.05), made no differences between two plants for locally rare fungal richness, and whole fungal richness (ANOVA *p* > 0.05) compared to monoculture. Intercropping increased the whole bacterial richness and Shannon diversity of the *Z. mays* rhizosphere but decreased the whole bacterial Shannon diversity, whole fungal Shannon and Simpson diversity, locally abundant fungal richness and Simpson diversity, and regionally abundant fungal Shannon and Simpson diversity of the *Z. mays* rhizosphere. Intercropping decreased the whole fungal Shannon diversity, locally abundant fungal richness, and regionally abundant fungal richness and Shannon diversity of *S. davidii* rhizosphere (ANOVA *p* < 0.05, Supplementary Table 2). Intercropping made no differences between two plants for locally abundant fungal FUNGuild functional richness and regionally rare fungal FUNGuild functional Simpson diversity (ANOVA *p* > 0.05). Intercropping increased regionally rare fungal FUNGuild functional Shannon and Simpson diversity of *Z. mays* rhizosphere; however, intercropping decreased whole fungal FUNGuild functional Shannon diversity and whole bacterial Tax4Fun functional Shannon and Simpson diversity of the *Z. mays* rhizosphere, increased regionally rare fungal FUNGuild functional Simpson diversity of the *S. davidii* rhizosphere. Intercropping also decreased whole bacterial FAPROTAX functional Simpson diversity (ANOVA *p* < 0.05, Supplementary Table 2).

### Intercropping shifted the taxonomic and functional composition of rhizosphere microbes

**FIGURE 5:**
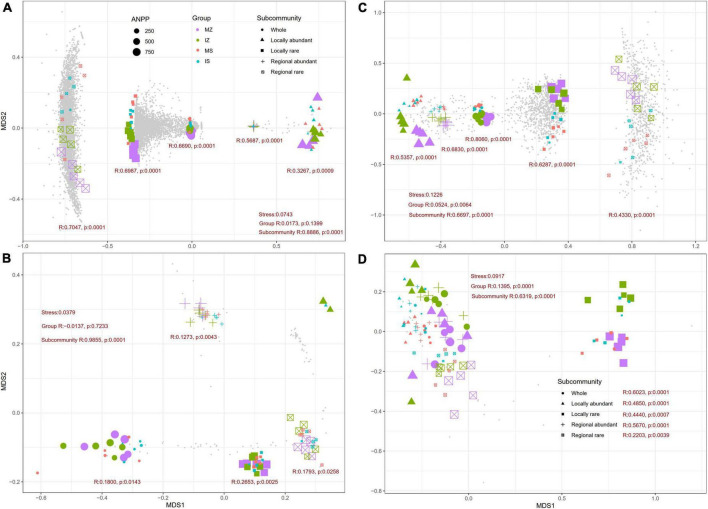
Non-metric multidimensional scaling (NMDS) ordinations based on Bray–Curtis distance matrices of taxonomy **(A,B)** and functions **(C,D)** of bacterial **(A,C)** and fungal **(B,D)** subcommunities for all samples (*n* = 20). A stress <0.1 indicated that the result of NMDS is reliable. ANPP, aboveground net primary productivity; MZ, monoculture *Zea mays*; IZ, intercropping *Zea mays*; MS, monoculture *Sophora davidii*; IZ, intercropping *Sophora davidii*. *R* and *p* were reported by ANOSIM.

The NMDS and ANOSIM test showed bacterial species, fungal species, and fungal functions significantly differed among groups in each subcommunity (*p* < 0.01, Figure 5), and bacterial functions significantly differed among groups in each subcommunity (*p* < 0.05), except for the locally abundant species subcommunity. LEfSe analysis showed that, in the *Z. mays* rhizosphere, four bacterial clades and 36 fungal phylotypes were evidently enriched under monoculture (*p* < 0.05, Supplementary Figures 3A,B), and 18 bacterial phylotypes and one fungal phylotype were evidently enriched under intercropping. In the *S. davidii* rhizosphere, 10 bacterial phylotypes and nine fungal phylotypes were evidently enriched under monoculture, and five bacterial phylotypes and four fungal phylotypes were evidently enriched under intercropping (*p* < 0.05, Supplementary Figures 3C,D). Across groups, one bacterial phylotype and two fungal phylotypes were evidently enriched in the *S. davidii* rhizosphere under monoculture, six bacterial phylotypes and one fungal phylotype were evidently enriched in the *S. davidii* rhizosphere under intercropping, two bacterial phylotypes and 13 fungal phylotypes were evidently enriched in the *Z. mays* rhizosphere under monoculture, and one bacterial phylotype and no fungal phylotype were evidently enriched in the *Z. mays* rhizosphere under intercropping (*p* < 0.05). We also examined any significant changes in species or function among groups using the Kruskal–Wallis rank sum test. A total of 1,383 bacterial species, 34 bacterial functions, 314 fungal species, and 16 fungal functions with significant changes were detected (*p* < 0.05), 0–50.0% of which were locally abundant species, 30.6–50.0% were locally rare species, 4.9–68.8% were regionally abundant species, and 8.3–29.4% were regionally rare species.

The networks of the rare subcommunity and whole community have higher complexity than that of the abundant (Supplementary Figures 4, 5). For *Z. mays*, intercropping increased the number of edges, vertices, and positive edges of whole, locally rare, regionally rare bacteria networks, but decreased those of locally abundant bacteria networks, and for *S. davidii*, intercropping decreased those of whole, locally abundant, locally rare, regionally rare bacteria networks. This indicated that the effect of intercropping on the network of bacterial subcommunity was regulated by plant species. However, consistent decline in the edges, vertices, and positive edges by intercropping were observed in most of fungal subcommunity, regardless of plant species (Table 1). These results indicated that intercropping increased the complexity and positive links of rare bacteria in *Z. mays* rhizosphere, but decreased the complexity and positive links of rare in *S. davidii* rhizosphere, and the complexity and positive links of fungi in both intercropped plants rhizosphere.

**TABLE 1:** Properties of subcommunity microbial association network in different groups.

Subcommunity		Bacteria			Fungus		
			
	Group	Edges	Vertices	Positive edges	Edges	Vertices	Positive edges
Whole	MZ	1211186	6334	1210331	55700	1126	55671
	IZ	1291873	6599	1291010	46500	1023	46451
	MS	1416159	6711	1416039	40500	952	40495
	IS	1059633	6045	1059470	35700	893	35743
Locally abundant	MZ	1	2	1	116	36	116
	IZ	0	0	0	25	16	25
	MS	5	7	5	28	19	28
	IS	3	3	3	25	15	25
Locally rare	MZ	1448544	7004	1448470	56200	1094	56219
	IZ	1506957	7256	1506866	52700	1078	52720
	MS	1674575	7396	1674539	47500	975	47516
	IS	1296898	6763	1294708	43700	957	43699
Regionally abundant	MZ	0	0	0	13	14	13
	IZ	0	0	0	13	8	13
	MS	0	0	0	2	4	2
	IS	1	2	0	3	3	3
Regionally rare	MZ	899325	4219	899325	19100	533	19118
	IZ	1030793	4473	1030793	17800	481	17823
	MS	1041628	4376	1041628	12400	418	12400
	IS	796753	3844	795265	11400	419	11400

MZ, monoculture Zea mays; IZ, intercropping Zea mays; MS, monoculture Sophora davidii; IS, intercropping Sophora davidii.

### Rhizosphere rare taxa were related to rhizosphere soil functions and aboveground net primary productivity, and changed trade-offs

**FIGURE 6:**
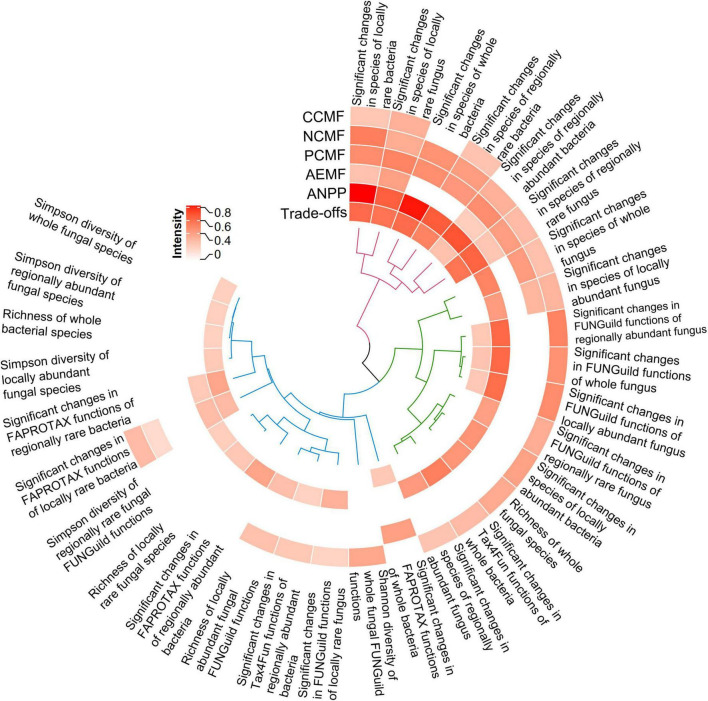
Heatmap showing the intensity of the selected species (or functions) or the diversity indices impacting rhizosphere functions and aboveground net primary productivity (ANPP). The intensity was indicated by *r* of Mantel test. The selected species (or functions) were the intersection of species or functions that significantly differed among the groups (MZ, IZ, MS, and IS) and were significantly correlated with system function (C/N/P-circling multifunctionality, AEMF, and ANPP, respectively). Only the significant intensity was showed. Hierarchical cluster analysis showed the clusters of factors. CCMF, carbon-circling multifunctionality; NCMF, nitrogen-circling multifunctionality; PCMF, phosphorus-circling multifunctionality; AEMF, average ecosystem multifunctionality.

The Spearman correlation analysis showed different species, functions or diversity indices were significantly related to CCMF, NCMF, and PCMF, AEMF, and ANPP (*p* < 0.05), respectively. Mantel test and Hierarchical cluster analysis showed three clusters of factors that can impact the system functions (Figure 6). A higher r indicates a stronger relationship. Significant changes in species of locally rare bacteria (Mantel *r* = 0.8185, *p* = 0.0001) had a stronger relationship with ANPP than others did (Mantel *r* = 0.2107 – 0.8024, *p* = 0.0001–0.0228, Figure 6). Significant changes in species of locally rare fungus (Mantel *r* = 0.5069, *p* = 0.0001) had a stronger relationship with PCMF than others did (Mantel *r* = −0.0127 – 0.4767, *p* = 0.0001 – 1). Significant changes in species of locally rare bacteria (Mantel *r* = 0.5340, *p* = 0.0001) had a stronger relationship with NCMF than others did (Mantel *r* = 0 – 0.5118, *p* = 0.0001 – 1). Significant changes in species of locally rare fungus (Mantel *r* = 0.3295, *p* = 0.009) had a stronger relationship with CCMF than others did (Mantel *r* = −0.0800 – 0.2890, *p* = 0.0092 – 1). Significant changes in species of locally rare fungus (Mantel *r* = 0.3984, *p* = 0.0017) had a stronger relationship with AEMF than others did (Mantel *r* = 0.0079 – 0.2604, *p* = 0.0151 – 1). Significant changes in species of locally rare bacteria (Mantel *r* = 0.6026, *p* = 0.0001) had a stronger relationship with trade-offs intensity (Mantel *r* = 0.1983 – 0.5962, *p* = 0.0001 – 1). The significant changes in rhizosphere species and functions had a stronger relationship with CCMF (Mantel *r* = 0.2776 vs. 0, Kruskal–Wallis rank sum test *p* = 0.0277), NCMF (Mantel *r* = 0.3478 vs. 0.3285, Kruskal–Wallis rank sum test *p* = 0.9199), PCMF (Mantel *r* = 0.4325 vs. 0, Kruskal–Wallis rank sum test *p* = 0.0119), AEMF (Mantel *r* = 0.2786 vs. 0, Kruskal–Wallis rank sum test *p* = 0.0277), ANPP (Mantel *r* = 0.5074 vs. 0.2904, Kruskal–Wallis rank sum test *p* = 0.0365), and trade-offs (Mantel *r* = 0.3931 vs. 0.2084, Kruskal–Wallis rank sum test *p* = 0.0036) than the significantly diversity indices.

## Discussion

### The selection effect was stronger than the complementary effect on multiple functions

In agreement with findings from macroecology (Karolína et al., 2014; Baldrighi et al., 2017; Fanin et al., 2017; Bagousse-Pingueta et al., 2019) and bulk soils (Bastida et al., 2016; Delgado-Baquerizo et al., 2016; Mori et al., 2016), the diversities of the microbes in the rhizosphere played an essential role in driving multiple functions (Supplementary Table 3). This indicated that maintaining functioning needs protection and use of rhizosphere microbial diversity. Furthermore, the positive effects dominated CCMF, but the negative effects dominated AEMF. The positive and negative effects on NCMF and ANPP were detected. The negative effects (negative correlation, 15/17) were more frequent than the positive effects (positive correlation, 2/17) in driving NCMF (Supplementary Table 3), indicating that the negative effects dominated the NCMF. However, the positive effects (positive correlation, 8/9) were more frequent than the negative effects (negative correlation, 1/9) in driving ANPP (Supplementary Table 3), suggested that the positive effects dominated ANPP.

Here, we showed for the first time that (Figure 5) the significant changes in rhizosphere species and functions had a stronger relationship with CCMF, NCMF, PCMF, AEMF, ANPP, and trade-offs than the significantly diversity indices, indicating that the selection effect played a chief role in driving multiple functions. This finding was different from the findings of studies in the macroecology (Tonin et al., 2018; Woodcock et al., 2019; Chun et al., 2020) and bulk soils (Wen et al., 2020), which indicated that the complementarity effect played a chief role in driving multiple functions whereas the selection effect had a limited role (Emily et al., 2018; Li et al., 2020a). This inconsistency indicated that the selection effect in the rhizosphere could not be ignored and might be a novel way to regulate productivity. Our result could be explained by the following potential causes: (1) The rhizosphere community was under strong selective pressure for special microbes (Poole et al., 2018) based on crucial functions associated with the metabolism of N and P, which are associated with plant growth promotion and nutrition (Mendes et al., 2014), therefore, plant productivity relied on the microbiome for the uptake of nutrients (Chen et al., 2020b). (2) The rhizosphere microbes mediated the root exudation (Korenblum et al., 2020), changed the nutrient supply and absorption of plants and rhizosphere NCMF. In turn, rhizosphere NCMF negatively modulated plant productivity (Spearman *r* = −0.70, *p* < 0.001, Supplementary Figure 2). Collectively, the strong selectivity of plants to rhizosphere microorganisms (Philippot et al., 2013) determined which microorganisms or functions appear; therefore, the multiple functions of the rhizosphere and ANPP were more dependent on the selection effect than the complementary effect, indicating that community assembly processes might determine the diversity and composition of community, and it was the result of these processes that determined how selection and complementary effects occur (Leibold et al., 2017). Therefore, our study provided new insight into the rhizosphere that differ from bulk soils.

### Rare rhizosphere taxa might contribute over proportionately to multiple functions

Consistent with our previous findings from bulk soils (Ding and Wang, 2021), distinct microbes (bacteria or fungi) dominated the given functions. In this study, fungi likely dominated the CCMF and PCMF, bacteria likely dominated the NCMF and ANPP (Figure 6). This might be due to bacteria and fungus had different metabolic niches (Ding and Wang, 2021). For instance, fungus rather than most bacteria could secrete lignin-degrading enzymes (Ding et al., 2020a), enhanced rhizodeposition, and suppressed the organic matter degradation (Zhou et al., 2020a). Fungi dominated P uptake in symbiotic plants (Wang et al., 2020a; Jiang et al., 2021). The denitrification, mineralization, and assimilation of N were mainly driven by bacteria (Starke et al., 2016; Li et al., 2019; Ding and Wang, 2021). N is the major nutrient limiting plant growth (Moreau et al., 2019; Wang et al., 2020a), thus, if bacteria dominated the NCMF, bacteria were expected to dominate ANPP (Figure 6). Furthermore, this was the first study to report on locally rare bacteria and fungi were overlooked keystone taxa shaping ecosystem functions and trade-offs. These findings supported established results (Radchuk et al., 2016; Jousset et al., 2017; Dee et al., 2019; Zhang et al., 2019; Chen et al., 2020a).

Rare taxa could act as keystone species through several mechanisms. We grouped the potential mechanisms accounting for the greater contribution of the rare species to ecosystem functions into three pathways that may operate simultaneously: (1) Rare species occupied the majority of species in ecosystems (Jain et al., 2014; Lynch and Neufeld, 2015; Jousset et al., 2017; Zhang et al., 2019; Guo et al., 2020) and had extremely high diversity (Mo et al., 2018). Rare taxa had more stronger impact on the multifaceted diversity of the community than abundant taxa (Zhou et al., 2019a). In this study, the species richness of locally rare bacteria was 433- to 677-fold that of locally abundant bacteria, the species richness of regionally rare bacteria was six–seven fold that of regionally abundant bacteria, the species richness of locally rare fungus was 20- to 52-fold that of locally abundant fungus, the species richness of regionally rare bacteria was 1.2- to 1.8- fold that of regionally abundant fungus (Supplementary Table 2). On the one hand, rare species provided insurance effects (Jousset et al., 2017; Chen et al., 2020a) as implied by the insurance hypothesis (Jiao et al., 2017), and recruitment from the persistent rare microbial seed bank provided a broad reservoir of ecological function (Lynch and Neufeld, 2015). On the other hand, most distinct traits combinations were supported predominantly by rare species (David et al., 2013). Similar observations were detected in our study. Both locally and regionally, the function richness of rare bacteria and fungus occupied 57–100% of that of whole bacteria and fungus (Supplementary Table 2). According to the hypothesis of complementary and selection hypotheses (Mensah et al., 2020), high diversity elevated the chance of rare taxa to contributing to ecosystem functionality; in this sense, rare species had the potential to change an ecosystem’s multifunctionality via the enhancement of biodiversity (Angelini et al., 2015; Jousset et al., 2017). (2) Many studies had supported that “being different” was crucial for the influence of rare taxa/. In plant communities, rare species had a higher effect on ecosystem functioning because the of the rare taxa individual mass is higher than that of the abundant taxa (Radchuk et al., 2016). Similar effects had been observed in decomposition systems (Guo et al., 2020) and microbial system (Wei et al., 2019). Essentially, microbes changed system functions through their metabolic functions (Ding and Wang, 2021). In this study, there were obvious differences in functions (Figures 5C,D) and functional diversity (Supplementary Table 2) of the rare versus abundant species, suggested that rare species had the potential to act significant roles in ecosystem functioning via providing different functions. (3) Affecting the interactions. The effect of some taxa on ecosystem functions were not independent from their interactions with other taxa (Wagg et al., 2019). The disappearance of rare caused an obvious or bad alteration in the composition or function of the community (Nannipieri et al., 2020). In contrast, the occurrence of rare could have a good effect on the community (Xue et al., 2020). They could create circumstances that supported the co-occurrence of high densities of different functional organisms, thereby, enhancing MF (Angelini et al., 2015). Rare microbes could also heighten the role of abundant microbes (Jousset et al., 2017). Rare dissimilated low content compounds into materials needed by other microbes or synthesized effective bioactive compounds (Harrison et al., 2021). The rare could reshape rhizosphere community, thereby promote crop growth (Li et al., 2020b). Rare taxa could affect species interactions (Xue et al., 2020). In this study, rare contributed more to the microbe’s positive interaction than abundant (Table 1 and Supplementary Figures 4, 5). Higher frequent facilitations than competitions possibly yielded the complementarity effects (Ding and Wang, 2021). Therefore, rare contributed to ecosystem functions through species interactions (Jousset et al., 2017; Dee et al., 2019). Since rare species are most vulnerable to be lost (Dee et al., 2019) and are largely unexplored, we ascribed great importance to the rare species and suggested to optimize their taxa for maintaining a high ecosystem functionality.

### Rare rhizosphere taxa might contribute to trade-offs of multiple functions

Monoculture had higher ANPP than intercropping, this implied that monoculture brought higher benefits than intercropping, which was consistent with the recent findings in tropics (Grass et al., 2020). However, intercropping also shifted the ANPP-CCMF relationship from being none to being positive for *Z. mays* (Figures 2B,C), shifted the ANPP-NCMF relationship from being negative to being positive for *S. davidii* (Figures 2D,E). Intercropping decreased the trade-offs intensity compared to sole for *Z. mays* (Figure 3E). MF and yields were not always synergistic (Supplementary Figure 2), confirming recent findings (Garland et al., 2021). Our study also suggested, for the first time, that the locally rare bacteria species were most strongly related to the trade-offs of multiple functions, and indicated that the trade-offs would likely be reduced by optimizing the taxa of locally rare bacteria.

## Conclusion

This study investigated the rhizosphere abundant and rare bacteria and fungi, rhizosphere C/N/P-cycling multifunctionality, ecosystem multifunctionality, aboveground net primary productivity and trade-offs in the *Z. mays* and *S. davidii* sole and *Z. mays/S. davidii* intercropping ecosystems. Results demonstrated that intercropping altered multiple ecosystem functions individually and simultaneously. Intercropped *Z. mays* significantly decreased the trade-off intensity compared to sole *Z. mays*, the trade-off intensity under intercropped *S. davidii* was significantly higher than under intercropped *Z. mays*. Moreover, both rhizosphere abundant and rare could predict and might affect rhizosphere elements circling, multifunctionality, aboveground productivity and trade-offs, whereas, the significant changes in species of locally rare microbes were the best predictor of rhizosphere elements circling, multifunctionality, aboveground productivity and trade-offs. We thus ascribe great importance to the rare species. Indeed, our results may help in driving a high functionality by directing future efforts on collection, conservation and manipulation of rhizosphere rare species. Further research with more ecosystems and operation of rare microbe combinations will facilitate to maintain a higher ecosystem function and a better understanding of cause-and-effect mechanisms.

## Data availability statement

The datasets presented in this study can be found in online repositories. The names of the repository/repositories and accession number(s) can be found in the article/Supplementary material.

## Author contributions

PW and LD: conceptualization. PW: methodology and writing—original draft preparation. LD: data curation and visualization. CZ, LD, YZ, PW, and MW: investigation and writing—reviewing and editing. All authors contributed to the article and approved the submitted version.

## Footnotes

1
https://github.com/OpenGene/fastp

2
http://ccb.jhu.edu/software/FLASH/

3
https://www.mothur.org/

4
http://drive5.com/usearch

5
https://www.arb-silva.de/

6
http://unite.ut.ee

7
http://www.omicsmart.com

8
https://cran.r-project.org/bin/windows/base/old/3.5.3/R-3.5.3-win.exe

9
https://gephi.org/
