# Supplementary material for: Rhizosphere element circling, multifunctionality, aboveground productivity and trade-offs are better predicted by rhizosphere rare taxa
Source: Front Plant Sci. 2022 Sep 8;13:985574. doi: 10.3389/fpls.2022.985574 (PMC9495442; doi:10.3389/fpls.2022.985574)
Supplement: Supplementary file 1 [file Presentation_1.zip › Supplementary description of Method.docx]

**Rhizosphere element circl****ing, multifunctionality, aboveground productivity and trade-offs are better predicted by rhizosphere rare taxa**

**Puchang Wang^1†^, Leilei Ding^2†*^, Chao** **Zou^3^, Yujun Zhang^2^, & Mengya Wang^3^**

^1^School of Life Sciences, Guizhou Normal University, Guiyang 550025, Guizhou, China

^2^Guizhou Institute of Prataculture, Guizhou Academy of Agricultural Sciences, Guiyang 550006, Guizhou, China

^3^College of Animal Science, Guizhou University, Guiyang 550006, Guizhou, China

^†^The two authors contributed equally to this article and share first authorship.

*Corresponding author e-mail: peterding2007gy@163.com

**Supplementary description of Method**

***1*** ***Individual rhizosphere functions***

The rhizosphere physicochemical properties and C, N, and P-circling enzyme activities were assayed using the methods listed in previous studies (Bao 2000, Ding et al 2020a, Ding et al 2020b, Zheng 2019). Rhizosphere soil pH was measured with a suspension (soil: water=1:2.5 w/v); Water content (WC, %) was obtained by oven-drying at 105 ◦C (Ding et al 2020a). Rhizosphere C-circling functions were characterized as follows (Bastida et al 2016, Bowker et al 2013, Ding et al 2020a, Luo et al 2018): organic carbon (g kg^–1^) was assayed using potassium dichromate volumetric method, β-glucosidase (C-circling enzyme, μmol d^–1^ g^–1^ dry soil) was detected using an ELISA test kit (Shanghai Enzyme-linked Biotechnology Co., Ltd., China). Microbial biomass carbon (mg kg^–1^) was determined by the fumigation–extraction method. Rhizosphere N-circling functions were characterized as follows (Bagousse-Pingueta et al 2019, Ding et al 2020a, Jiao et al 2018, Luo et al 2018): ammoniacal nitrogen (mg kg^–1^) was assayed using ultraviolet spectrophotometry, nitrate nitrogen (mg kg^–1^) was using the colorimetric method based on extraction of potassium chloride, inorganic nitrogen (mg kg^–1^) was the sum of ammoniacal and nitrate nitrogen, five N-circling enzymes (N-acetylglucosaminidase; Leucine aminopeptidase, Nitrogenase, Nitric oxide synthetase, Glutamine synthetase; μmol d^–1^ g^–1^ dry soil) were detected using ELISA kits. Microbial biomass nitrogen (mg kg^–1^) was determined by the fumigation–extraction method. Rhizosphere P-circling functions were characterized as follows (Bagousse-Pingueta et al 2019, Bastida et al 2016, Bowker et al 2013, Ding et al 2020a, Jiao et al 2018, Luo et al 2018): total phosphorus (TP, mg kg^–1^) was determined by NaOH digestion, available phosphorus (AP, mg kg^–1^) was assayed via the NaHCO_3_-ultraviolet spectrometer, and acid phosphatase (P-circling enzyme, μmol d^–1^ g^–1^ dry soil) was detected using ELISA test kits.

Rhizosphere C-circling functions were characterized as follows (Bastida et al 2016, Bowker et al 2013, Ding et al 2020a, Luo et al 2018): organic carbon (OC, g/kg), β-glucosidase (C-circling enzyme, βG, μmol d^–1^ g^–1^), microbial biomass carbon (MBC, mg/kg). Rhizosphere N-circling functions were characterized as follows (Bagousse-Pingueta et al 2019, Ding et al 2020a, Jiao et al 2018, Luo et al 2018): ammoniacal nitrogen (NH4_N, mg/kg), nitrate nitrogen (NO3_N, mg/kg), inorganic nitrogen (IN, mg/kg), five N-circling enzymes (N-acetylglucosaminidase (NAG); Leucine aminopeptidase (LAP), Nitrogenase, Nitric oxide synthetase (NOS), Glutamine synthetase (GS); μmol d^–1^ g^–1^), microbial biomass nitrogen (MBN, mg/kg). Rhizosphere P-circling functions were characterized as follows (Bagousse-Pingueta et al 2019, Bastida et al 2016, Bowker et al 2013, Ding et al 2020a, Jiao et al 2018, Luo et al 2018): total phosphorus (TP, mg/kg), available phosphorus (AP, ug/kg), and acid phosphatase (P-circling enzyme, ACP, μmol d^–1^ g^–1^). Besides, rhizosphere soil pH and water content (WC, %) were detected. These properties were used because they either measure real functions or are good surrogates of element cycling (Bagousse-Pingueta et al 2019, Bowker et al 2013, Chen et al 2020a, Chen et al 2020b, Delgado-Baquerizo et al 2017).

***2*** ***DNA extraction,*** ***sequencing of 16S rRNA gene and ITS gene amplicons, and processing data***

Community DNA was extracted using HiPure Soil DNA Kits (Guangzhou Meiji Biotechnology Co., Ltd., China). The universal primers 341F (5′-CCTACGGGNGGCWGCAG-3′) and 806R (5′-GGACTACHVGGGTATCTAAT-3′) were used when the V3-V4 region of the 16S rRNA gene was PCR-amplified, and the universal primers ITS3_KYO2 (5′-GATGAAGAACGYAGYRAA-3′) and ITS4 (5′TCCTCCGCTTATTGATATGC-3′) were used when the internal transcribed spacer (ITS2) region of the fungal rRNA gene was PCR-amplified. The PCR procedure was conducted as follows: 94 °C for 2 min, followed by 30 cycles at 98 °C for 10 s, 62 °C for 30 s（except for 16S V4: 55°C for 30 s）, and 68 °C for 30 s and a final extension at 68 °C for 5 min. The PCR procedure was performed with a 50 μL mixture containing 5 μL of 10 × KOD Buffer, 5 μL of 2.5 mM dNTPs, 1.5 μL of each primer (5 μM), 1 μL of KOD Polymerase, and 100 ng of template DNA. FASTP (V 0.18.0, https://github.com/OpenGene/fastp) was used to removing reads containing more than 10% of unknown nucleotides and removing reads containing less than 50% of bases with quality (Q-value)＞20. Flash (V 1.2.11, http://ccb.jhu.edu/software/FLASH/) was used to merged pairs of double-ended reads into a sequence with a minimum overlap of 10 bp and a maximum mismatch rate of 2%. Mothur (v1.39.1, https://www.mothur.org/) was used to select the unique tag sequence. UPARSE (usearch v9.2.64, http://drive5.com/usearch) was used to obtain operational taxonomic unit (OTU) matrices at a 97% similarity threshold (Ding et al 2020a, Ding and Wang 2021). Sequencing of the 16S rRNA gene and ITS gene amplicons was performed using an Illumina platform, and bioinformatics analysis was done using an online platform (http://www.omicsmart.com) by Guangzhou Genedenovo Biotechnology Co., Ltd. (Guangzhou, China).

***3 Statistical analyses***

The differences and significance were tested using the work flow and methods in previous studies (Ding et al 2020a, Ding and Wang 2021). The ηp^2^ (partial eta-squared) statistic was conducted to test the relative influences of plant identity (*Sophora davidii* and *Zea mays*), system type (sole and intercropping) and interaction on each individual rhizosphere functions in IBM SPSS (version 25, IBM, Armonk, NY, USA). Principal component analysis (PCA) (Merino-Martín et al 2021) was used to determine which functions are the main ecosystem functions in the rhizosphere. The ANOSIM (Analysis of Similarities) test was used to determine significant differences in rhizosphere ecosystem functions between plant species (*Sophora davidii* and *Zea mays*), system type (sole and intercropping) and among groups (MZ, IZ, MS, and IS) and in species/functions among groups for each subcommunity. Correlation relationships among individual rhizosphere ecosystem functions were visualized based Spearman using “igraph” package (Csardi and Nepusz 2006).To retain the number of edges, vertices, and positive edges of microbial association networks, robust correlations were built based absolute value of Pearson correlation’s *r* > 0.8 and false discovery rate-corrected *p* < 0.001 using “WGCNA” and “igraph” packages in R. Gephi 0.9.2 (https://gephi.org/) were applied to visualize the networks.

Nonmetric multidimensional scaling (NMDS) (Jiao et al 2017) was applied to simplify samples or OUTs in high-dimensional to low-dimensional space for location using the “metaMDS” function based on the Bray-Curtis distance. Linear discriminant analysis (LDA) effect size (LEfSe) was run to determine the significantly enriched clades (LDA scores ≥ 2 and *p* < 0.05).

According to the definition of the selection effect (Mensah et al 2020), we used Spearman rank correlations (Zheng 2019) to select the species, functions, and diversity of the whole community, and abundant and rare subcommunities that were significantly related to C, N, P-circling multifunctionality (CCMF, NCMF, PCMF), average ecosystem multifunctionality (AEMF), and aboveground net primary productivity (ANPP), respectively. We used Kruskal-Wallis rank sum test to select the species, functions, and diversity of the whole, abundant, and rare subcommunities that significantly changed among groups. We then calculated the intersection of species/functions/diversities that significantly changed and were significantly related to the above ecosystem functions. To decipher how microbes link multifunctionality (Delgado-Baquerizo et al 2017), the Mantel test (Ding et al 2020a) with 9999 permutations was performed to detect the correlation between the intersection and above ecosystem functions, based on the Spearman method and Euclidean distance matrix. The greater the correlation of the Mantel test, the greater the impact intensity of species/functions/diversities on the above ecosystem functions (Ding and Wang 2021, Jiao et al 2017, Mo et al 2018, Xiong et al 2020, Xue et al 2020, Zheng 2019, Zhou et al 2019). The “ggplot2” (Wickham 2016) and “circlize” (Gu 2014) packages were used to visualize the results.

References

Bagousse-Pingueta YL, Soliveresc S, Grossa N, Toricesa R, Berdugoa M, Maestre FT (2019). Phylogenetic, functional, and taxonomic richness have both positive and negative effects on ecosystem multifunctionality. *PNAS* **116:** 8419-8424.doi:10.1073/pnas.1815727116

Bao D (2000). Soil and agricultural chemistry analysis, 3rd ed. *China Agriculture Press, Beijing***:** 14-210

Bastida F, Torres IF, Moreno JL, Baldrian P, Ondoño S, Ruiz‐Navarro A *et al* (2016). The active microbial diversity drives ecosystem multifunctionality and is physiologically related to carbon availability in Mediterranean semi‐arid soils. *Mol Ecol* **25:** 4660-4673.doi:10.1111/mec.13783

Bowker MA, Maestre FT, Mau RL (2013). Diversity and Patch-Size Distributions of Biological Soil Crusts Regulate Dryland Ecosystem Multifunctionality. *Ecosystems* **16:** 923–933.doi:10.1007/s10021-013-9644-5

Chen QL, Ding J, Zhu D, Hu HW, Delgado-Baquerizo M, Ma YB *et al* (2020a). Rare microbial taxa as the major drivers of ecosystem multifunctionality in long-term fertilized soils. *Soil Biol Biochem* **141:** 107686.doi:10.1016/j.soilbio.2019.107686

Chen QL, Ding J, Zhu YG, He JZ, Hu HW (2020b). Soil bacterial taxonomic diversity is critical to maintaining the plant productivity. *Environ Int* **140:** 105766.doi:10.1016/j.envint.2020.105766

Csardi G, Nepusz T (2006). The igraph software package for complex network research. *InterJournal Complex Systems***:** 1695.doi:<http://igraph.org>

Delgado-Baquerizo M, Trivedi P, Trivedi C, Eldridge DJ, Reich PB, Jeffries TC *et al* (2017). Microbial richness and composition independently drive soil multifunctionality. *Funct Ecol* **31:** 2330-2343.doi:10.1111/1365-2435.12924

Ding L, Shang Y, Zhang W, Zhang Y, Li S, Wei X *et al* (2020a). Disentangling the effects of driving forces on soil bacterial and fungal communities under shrub encroachment on the Guizhou Plateau of China. *Sci Total Environ* **709:** 136207.doi:10.1016/j.scitotenv.2019.136207

Ding L, Wang P, Zhang W, Zhang Y, Li S, Wei X *et al* (2020b). Soil stoichiometry modulates effects of shrub encroachment on soil carbon concentration and stock in a subalpine grassland. *iForest Biogeosci For* **13:** 65-72.doi:10.3832/ifor3091-012

Ding L, Wang P (2021). Afforestation suppresses soil nitrogen availability and soil multifunctionality on a subtropical grassland. *Sci Total Environ* **761:** 143663.doi:10.1016/j.scitotenv.2020.143663

Gu Z (2014). circlize implements and enhances circular visualization in R. *Bioinformatics* **30:** 2811-2812.doi:10.1093/bioinformatics/btu393

Jiao S, Luo Y, Lu M, Xiao X, Lin Y, Chen W *et al* (2017). Distinct succession patterns of abundant and rare bacteria in temporal microcosms with pollutants. *Environ Pollut* **225:** 497-505.doi:10.1016/j.envpol.2017.03.015

Jiao S, Chen W, Wang J, Du N, Li Q, Wei G (2018). Soil microbiomes with distinct assemblies through vertical soil profiles drive the cycling of multiple nutrients in reforested ecosystems. *Microbiome* **6:** 1-13.doi:10.1186/s40168-018-0526-0

Luo G, Rensing C, Chen H, Liu M, Wang M, Guo S *et al* (2018). Deciphering the associations between soil microbial diversity and ecosystem multifunctionality driven by long‐term fertilization management. *Funct Ecol* **32:** 1103-1116.doi:10.1111/1365-2435.13039

Mensah S, Kolawolé Valère Salako, Assogbadjo A, Romain Glèlè Kaka, Brice， S, Seifert T (2020). Functional trait diversity is a stronger predictor of multifunctionality than dominance: evidence from an afromontane forest in south africa. *Ecol Indic* **115:** 106415.doi:10.1016/j.ecolind.2020.106415

Merino-Martín L, Stokes A, Gweon HS, Moragues-Saitua L, Staunton S, Plassard C *et al* (2021). Interacting effects of land use type, microbes and plant traits on soil aggregate stability. *Soil Biology and Biochemistry* **154**.doi:10.1016/j.soilbio.2020.108072

Mo Y, Zhang W, Yang J, Lin Y, Yu Z, Lin S (2018). Biogeographic patterns of abundant and rare bacterioplankton in three subtropical bays resulting from selective and neutral processes. *ISME J* **12:** 2198-2210.doi:10.1038/s41396-018-0153-6

Wickham H (2016). ggplot2: Elegant Graphics for Data Analysis. *Springer-Verlag New York***:** <https://ggplot2.tidyverse.org.doi:https://ggplot2.tidyverse.org>

Xiong C, He JZ, Singh BK, Zhu YG, Wang JT, Li PP *et al* (2020). Rare taxa maintain the stability of crop mycobiomes and ecosystem functions. *Environ Microbiol*.doi:10.1111/1462-2920.15262

Xue M, Guo Z, Gu X, Gao H, Weng S, Zhou J *et al* (2020). Rare rather than abundant microbial communities drive the effects of long-term greenhouse cultivation on ecosystem functions in subtropical agricultural soils. *Sci Total Environ* **706:** 136004.doi:10.1016/j.scitotenv.2019.136004

Zheng Q, Hu, Y. , Zhang, S. , Noll, L. , & Wanek, W. . (2019). Soil multifunctionality is affected by the soil environment and by microbial community composition and diversity. , 136, 107521-. *Soil Biol Biochem* **136:** 107521.doi:10.1016/j.soilbio.2019.107521

Zhou Y, Qin Y, Liu X, Feng Z, Zhu H, Yao Q (2019). Soil bacterial function associated with stylo (legume) and bahiagrass (grass) is affected more strongly by soil chemical property than by bacterial community composition. *Front Microbiol* **10:** 1-12.doi:10.3389/fmicb.2019.00798
