# Supplementary material for: Rhizosphere element circling, multifunctionality, aboveground productivity and trade-offs are better predicted by rhizosphere rare taxa
Source: Front Plant Sci. 2022 Sep 8;13:985574. doi: 10.3389/fpls.2022.985574 (PMC9495442; doi:10.3389/fpls.2022.985574)
Supplement: Supplementary file 1 [file Presentation_1.zip › Captions for Figure.S1-5.docx]

**Rhizosphere element circl****ing, multifunctionality, aboveground productivity and trade-offs are better predicted by rhizosphere rare taxa**

**Puchang Wang^1†^, Leilei Ding^2†*^, Chao** **Zou^3^, Yujun Zhang^2^, & Mengya Wang^3^**

^1^School of Life Sciences, Guizhou Normal University, Guiyang 550025, Guizhou, China

^2^Guizhou Institute of Prataculture, Guizhou Academy of Agricultural Sciences, Guiyang 550006, Guizhou, China

^3^College of Animal Science, Guizhou University, Guiyang 550006, Guizhou, China

^†^The two authors contributed equally to this article and share first authorship.

*Corresponding author e-mail: peterding2007gy@163.com

**The captions for Figure.S1-5**

**Figure. S1** Rhizosphere soil inorganic nitrogen content (IN, **a**) and pH (**b**) in different groups. “ns”, *p* > 0.05; MZ, Monoculture *Zea mays*; IZ, Intercropping *Zea mays*; MS, Monoculture *Sophora davidii*; IZ, Intercropping *Sophora davidii*.

**Figure. S2** Spearman correlation analysis showing the relationship between rhizosphere C, N, P circling multifunctionality (CCMF, NCMF, PCMF), average rhizosphere ecosystem multifunctionality (AEMF) and aboveground net primary productivity (ANPP). “*”, *p* < 0.05; “**”, *p* < 0.01; “***”, *p* < 0.001.

**Figure. S3** Linear discriminant analysis (LDA) indicating significantly enriched taxa (LDA scores ≥ 2 and *p* <0.05); MZ, Monoculture *Zea mays*; IZ, Intercropping *Zea mays*; MS, Monoculture *Sophora davidi*i; IS, Intercropping *Sophora davidii*. (**a, b, e**) Significantly enriched bacterial taxa among groups. (**c, d, f**) Significantly enriched fungal taxa among groups.

**Figure. S4** Association networks of each bacterial subcommunity in different groups (MZ, IZ, MS and IS) with colour vertices showing the eight main ecological modules. (**a**) whole communities. (**b**) regionally abundant subcommunities. (**c**) regionally rare subcommunities. (**d**) locally abundant subcommunities. (**e**) locally rare subcommunities. Circles indicate individual operational taxonomic units (OTUs). Red edges, positive correlation; Blue edges, negative correlation; MZ, Monoculture *Zea mays*; IZ, Intercropping *Zea mays*; MS, Monoculture *Sophora davidi*i; IS, Intercropping *Sophora davidii*.

**Figure. S5** Association networks of each fungal subcommunity in different groups (MZ, IZ, MS and IS) with colour vertices showing the eight main ecological modules. (**a**) whole communities. (**b**) regionally abundant subcommunities. (**c**) regionally rare subcommunities. (**d**) locally abundant subcommunities. (**e**) locally rare subcommunities. Circles indicate individual operational taxonomic units (OTUs). Red edges, positive correlation; Blue edges, negative correlation; MZ, Monoculture *Zea mays*; IZ, Intercropping *Zea mays*; MS, Monoculture *Sophora davidi*i; IS, Intercropping *Sophora davidii*.
