# Supplementary figures and images for: Rhizosphere element circling, multifunctionality, aboveground productivity and trade-offs are better predicted by rhizosphere rare taxa
Source: Front Plant Sci. 2022 Sep 8;13:985574. doi: 10.3389/fpls.2022.985574 (PMC9495442; doi:10.3389/fpls.2022.985574)

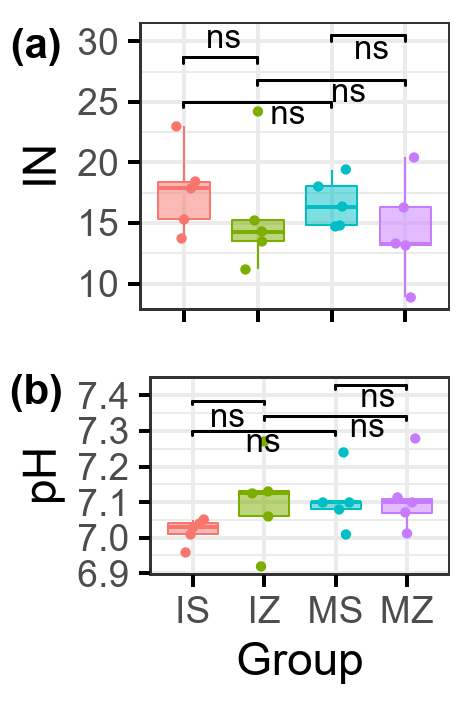

Supplement: Supplementary file 1 [file Presentation_1.zip › Figure.S1.tif]

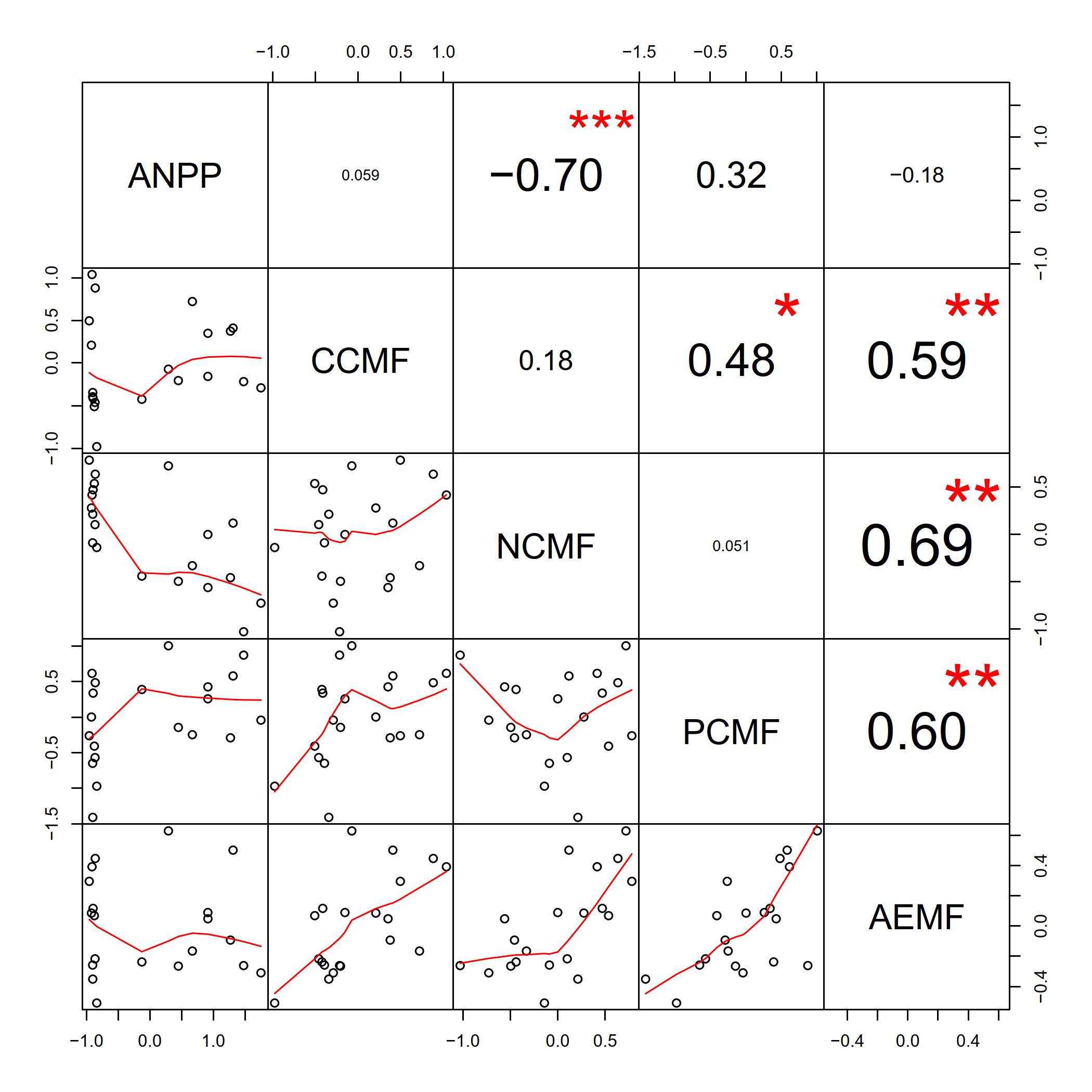

Supplement: Supplementary file 1 [file Presentation_1.zip › Figure.S2.tif]

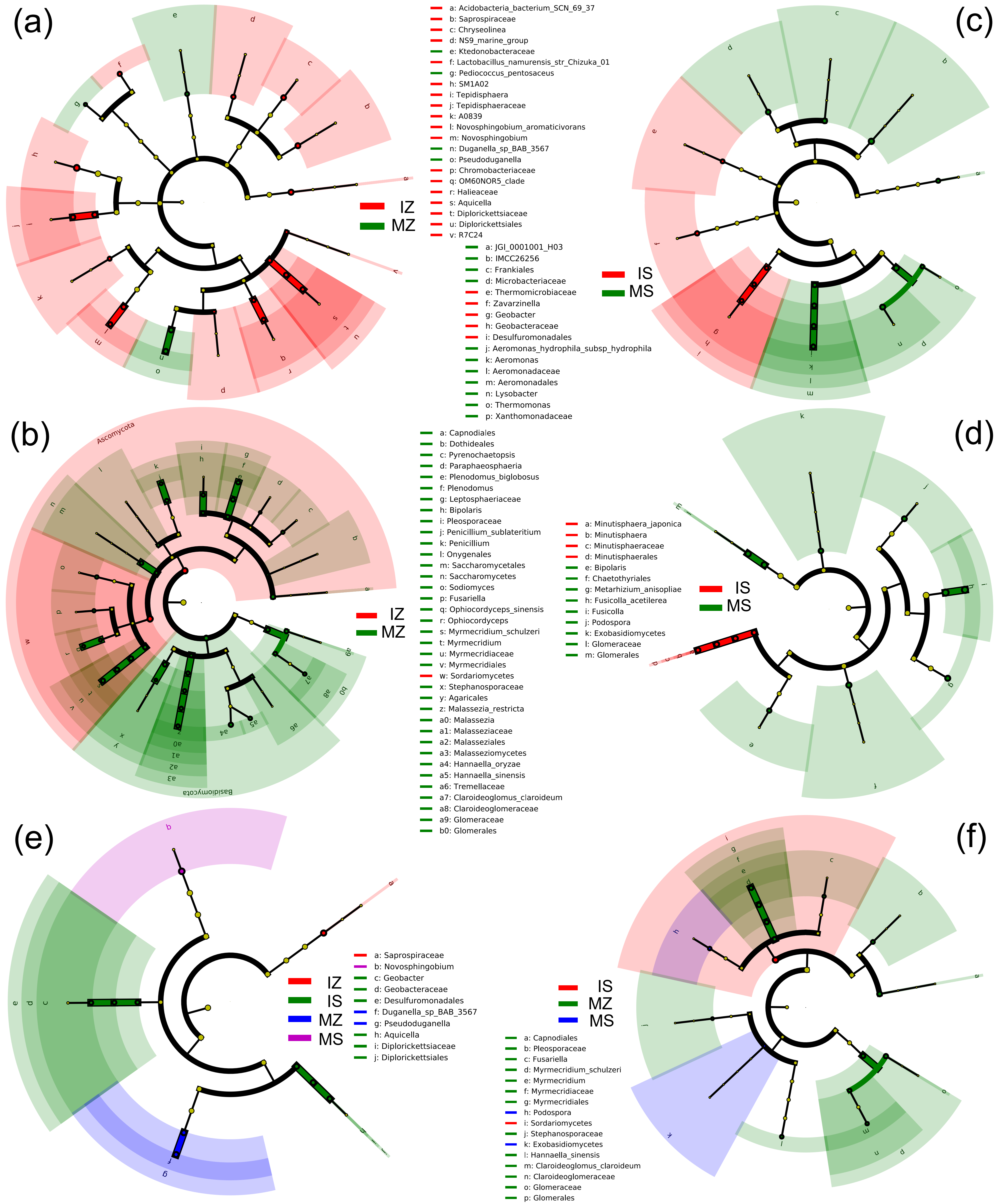

Supplement: Supplementary file 1 [file Presentation_1.zip › Figure.S3.tif]

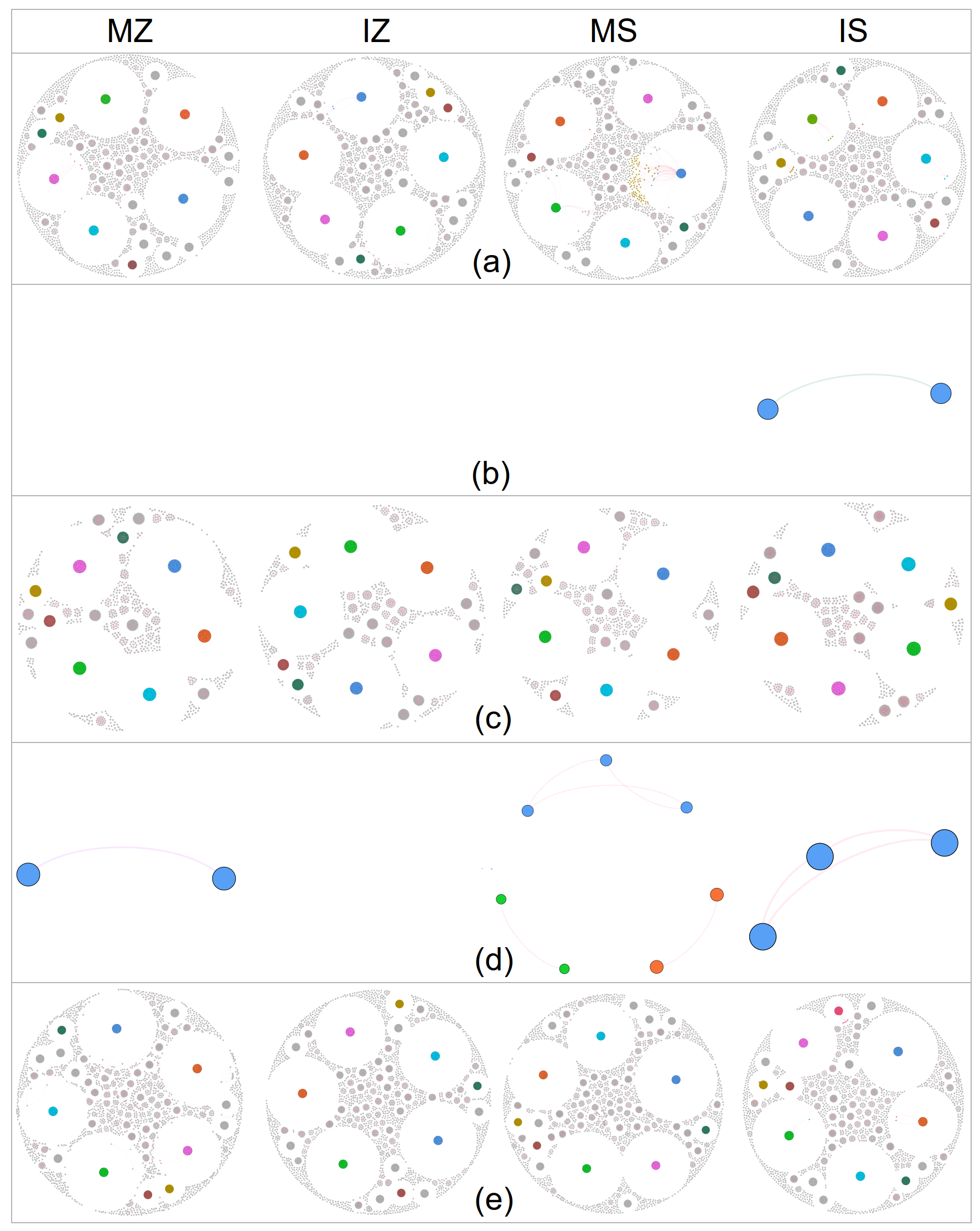

Supplement: Supplementary file 1 [file Presentation_1.zip › Figure.S4.tif]

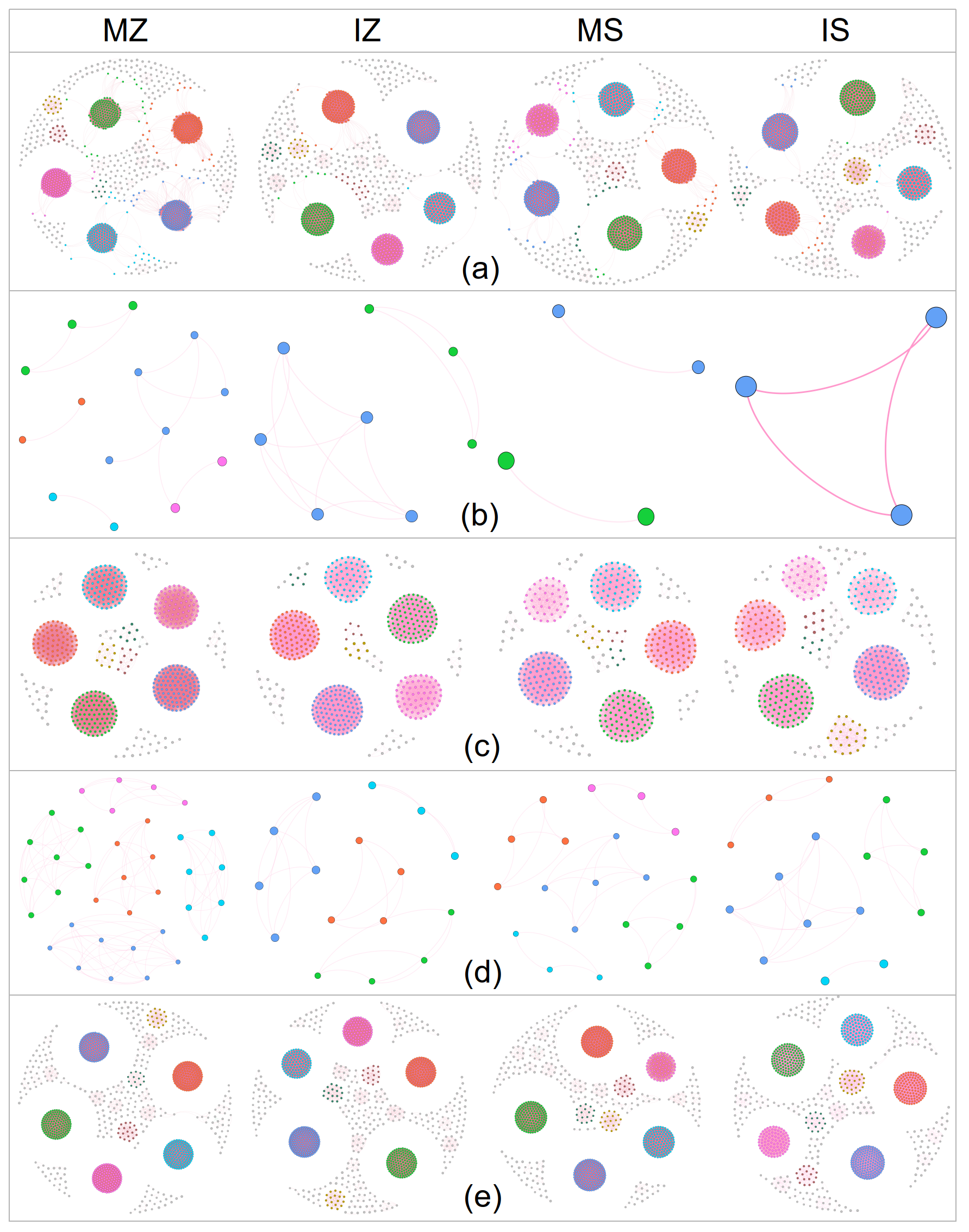

Supplement: Supplementary file 1 [file Presentation_1.zip › Figure.S5.tif]
